# Relationship between Cortical Thickness and Neuropsychological Performance in Normal Older Adults and Those with Mild Cognitive Impairment

**DOI:** 10.14336/AD.2018.0125

**Published:** 2018-12-04

**Authors:** Calvin Pak-Wing Cheng, Sheung-Tak Cheng, Cindy Woon-Chi Tam, Wai-Chi Chan, Winnie Chiu-Wing Chu, Linda Chiu-Wa Lam

**Affiliations:** ^1^Department of Psychiatry, Queen Mary Hospital, The University of Hong Kong, Hong Kong; ^2^Department of Health and Physical Education, The Education University of Hong Kong and Norwich Medical School, University of East Anglia, UK; ^3^Department of Psychiatry, North District Hospital, Hong Kong; ^4^Department of Psychiatry, Queen Mary Hospital, The University of Hong Kong, Hong Kong; ^5^Department of Imaging and Interventional Radiology, The Chinese University of Hong Kong, Hong Kong; ^6^Department of Psychiatry, Tai Po Hospital, The Chinese University of Hong Kong, Hong Kong

**Keywords:** cortical thickness, dementia, mild cognitive impairment, neuropsychological performance, magnetic resonance imaging

## Abstract

Mild cognitive impairment (MCI) has been extensively investigated in recent decades to identify groups with a high risk of dementia and to establish effective prevention methods during this period. Neuropsychological performance and cortical thickness are two important biomarkers used to predict progression from MCI to dementia. This study compares the cortical thickness and neuropsychological performance in people with MCI and cognitively healthy older adults. We further focus on the relationship between cortical thickness and neuropsychological performance in these two groups. Forty-nine participants with MCI and 40 cognitively healthy older adults were recruited. Cortical thickness was analysed with semiautomatic software, Freesurfer. The analysis reveals that the cortical thickness in the left caudal anterior cingulate (p=0.041), lateral occipital (p=0.009) and right superior temporal (p=0.047) areas were significantly thinner in the MCI group after adjustment for age and education. Almost all neuropsychological test results (with the exception of forward digit span) were significantly correlated to cortical thickness in the MCI group after adjustment for age, gender and education. In contrast, only the score on the Category Verbal Fluency Test and the forward digit span were found to have significant inverse correlations to cortical thickness in the control group of cognitively healthy older adults. The study results suggest that cortical thinning in the temporal region reflects the global change in cognition in subjects with MCI and may be useful to predict progression of MCI to Alzheimer’s disease. The different pattern in the correlation of cortical thickness to the neuropsychological performance of patients with MCI from the healthy control subjects may be explained by the hypothesis of MCI as a disconnection syndrome.

The significant growth in the population with dementia has been highlighted as a public health priority [1]. A wide range of cognitive impairment is the core symptom of dementia and determines the loss of independent functioning. Mild cognitive impairment (MCI) is a transitional state between normal ageing and dementia [2]. MCI has been extensively investigated in recent decades to identify those with a high risk of dementia and to establish effective prevention methods during this period. Neuropsychological performance and cortical thickness are two important biomarkers used to predict progression from MCI to dementia.

Sub-normative neuropsychological performance is one of the core diagnostic criteria for MCI. A wide range of cognitive impairment, including memory, attention and executive functions, can be found in patients with MCI. In addition to its diagnostic value, neuropsychological assessment also provides a possible means of differentiating high-risk groups for different types of dementia [3].

Along with the rapid development of neuroimaging techniques, the use of cortical thickness as measured on T1-weighted magnetic resonance imaging (MRI) as a biomarker to predict or facilitate early diagnosis of dementia has become a research direction of great interest. Compared to voxel-based morphology (VBM), the measurement of cortical thickness allows more precise measurement in deep sulci and analysis of the morphology as a cortical sheet [4]. Convergent findings strongly suggest a significant difference in cortical thickness amongst normal control patients, those with MCI and those with dementia [5-7]. Furthermore, a longitudinal study of 382 participants who were followed up for 24 months suggested that cortical thickness was sensitive for the early diagnosis of Alzheimer’s disease [8]. Another study reported that a decrease in cortical thickness could be detected in cognitively normal individuals several years before the onset of clinical symptoms [9].

Cortical thickness was suggested to have a close relationship with neuropsychological performance [10]. Despite the consistent evidence in support of this hypothesis, large variations were found across studies in the correlation of cortical thickness to neuropsychological performance amongst normal older adults and those with MCI and AD. Verbal memory performance was found to be associated with the medial temporal cortical thickness in normal subjects [11]. In subjects with MCI, the thickness of the entorhinal and praecuneus cortices predicted learning, whereas the posterior cingulate cortical thickness predicted learning in subjects with AD [12]. Another study suggested that MCI entails a specific cortical thinning relationship with high-level executive outcomes that is qualitatively different from that observed in healthy older adults [13]. This variation in the correlational patterns may shed light on the underlying differences in the cognitive processes and compensatory mechanisms between people with MCI and normal older adults. There is a paucity of research into differences between people with MCI and healthy subjects in the relationship between neuropsychological performance and cortical thickness. Therefore, we conducted this study to compare the cortical thickness and neuropsychological performance between subjects with MCI and healthy older adults. The relationship between the cortical thickness and neuropsychological performance in these two groups was also examined. We hypothesised that subjects with MCI would have thinner cortices and would display worse neuropsychological performance than healthy older adults. The correlation between the brain cortical thickness and a specific neuropsychological performance may have different patterns in these two groups.

## MATERIALS AND METHODS

### Subjects

Forty-nine patients with MCI and 40 cognitively healthy elderly control subjects (healthy controls; HC) were recruited. All of the participants were recruited from local elderly community centres. The study was approved by the Clinical Research Ethics Committee of The Chinese University of Hong Kong (NTEC-CUHK ethics committee). Written informed consent was obtained from all of the participants.

All of the participants underwent a battery of neuropsychological tests to evaluate their cognitive functions.

The Cantonese version of the Mini-Mental State Examination (CMMSE) [14, 15] was used to evaluate general cognitive function. The Clinical Dementia Rating (CDR) [16] scale was used to measure the severity of dementia. The Chinese version of the Alzheimer’s Disease Assessment Scale-Cognitive Subscale (ADAS-Cog) [17, 18] was used to assess the global cognitive deficit in patients with MCI. In addition, the forward and backward digit span tests from the Wechsler Adult Intelligence Scale [19] were used to assess the function of short-term memory and working memory, respectively. The Category Verbal Fluency Test (CVFT) [20, 21] was used to examine executive and semantic memory functions. The diagnosis of MCI was made by expert neurologists based on the Mayo Clinic Criteria [2], which includes (1) subjective memory complaints, (2) objective memory impairment (i.e., delayed recall scores of at least 1.5 standard deviations below age- and education-matched persons with a CDR of 0), (3) intact daily life activities, (4) a CDR score of 0.5 and (5) no clinical dementia (CMMSE score > 22 for older adults with more than 2 years of education, CMMSE score > 20 for older adults with less than 2 years of education and CMMSE score > 19 for older adults with no education [22]. Participants with profound sensory deficits or psychiatric (i.e., dependence on alcohol or other substances) and/or neurological disorders other than dementia (i.e., head trauma, multiple sclerosis and Parkinson’s disease) were excluded.

### MRI acquisition

The MRI images were acquired using a 3 Tesla Philips MRI scanner (Achieva TX, Philips Medical Systems, Best, the Netherlands) with an eight-channel SENSE head coil. A 3D high-resolution T1-weighted anatomical image was obtained for each participant (repetition time [TR] = 7.4 ms; echo time [TE] = 3.4 ms; flip angle = 8°; voxel size = 1.04 × 1.04 × 0.6 mm^3^).

### Cortical thickness analysis

The image data were exported from the MRI scanner to a personal computer for morphometric analysis. Before analysis, all images were checked for severe head motion. Semi-automatic software, the FreeSurfer version 5.3 software package (http://surfer.nmr.mgh.harvard.edu), was used to obtain estimates of cortical thickness, which was measured by reconstructing representations of the grey/white matter boundary and the cortical surface and then calculating the distance between those surfaces at numerous points (vertices) across the cortical mantle [23, 24]. Failures in FreeSurfer’s initial Talairach alignments were identified by visual inspection of all images and were rectified before reconstruction of the cortical surfaces. Topological defects in the automatically determined grey/white matter boundary were manually corrected. The cortical thickness values of 68 structures based on the Desikan-Killiany atlas were extracted from FreeSurfer [25]. All analyses were performed without knowledge of the subjects' identity.

### Statistical analysis

Linear regression adjusted for age and education was used for statistical analyses of the mean cortical thickness of region of interests (ROIs) between the subjects in the MCI and normal control groups, and p values of less than 0.05 were considered to indicate statistical significance. Partial correlations between neuropsychological scores and mean cortical thickness, adjusted for age, sex and years of education, were calculated for both MCI and control groups. Bonferroni correction was applied to correct for multiple comparisons, and p values of less than 0.01 were considered to indicate statistical significance after correction.

**Table 1:** Participant demographics and neuropsychological performance.

	Healthy Controls (n=40) Mean (SD)	MCI (n=49) Mean (SD)	p-value
Age	69.45 (4.56)	75.92 (5.39)	<0.001
Gender (Male: Female)	15:25	26:23	0.143
Education (years)	8.00 (4.00)	4.13 (4.04)	<0.001
CMMSE	27.68 (2.51)	24.94 (2.85)	<0.001
CDR - sum of boxes	0.16 (0.43)	1.02 (1.04)	<0.001
ADAS-Cog	6.46 (2.57)	13.59 (3.61)	<0.001
Delayed recall	6.58 (1.47)	2.29 (1.46)	<0.001
CVFT	40.10 (7.58)	31.27 (8.03)	<0.001
Digit span test (forward)	7.50 (1.36)	6.80 (1.44)	0.021
Digit span test(backward)	3.93 (1.65)	2.59 (1.39)	<0.01

ADAS-Cog - Chinese version of the Alzheimer’s Disease Assessment Scale–Cognitive Subscale; CDR - Clinical Dementia Rating; CMMSE - Cantonese version of the Mini-Mental State Examination; CVFT - Category Verbal Fluency Test

## RESULTS

### Demographic and baseline data

Table 1 shows significant differences in age and education between the MCI group and the HC group. Compared with those with MCI, the participants in the HC group were younger (mean [SD], 69.45 [4.56] vs. 75.92 [5.39]) and had more years of education (mean [SD], 8.00 [4.00] vs. 4.13 [4.04]). No significant difference was found in the gender ratio. The participants with MCI had significantly lower scores on the CMMSE, CDR sum of boxes, ADAS-Cog, CVFT and forward and backward digit span tests than the subjects in the HC group (p<0.05). The mean CMMSE score in the MCI group was 24.94, and that in the HC group was 27.68.

### Difference in cortical thickness between MCI and HC groups

The mean cortical thicknesses of all areas in the brain are shown in Table 2 for the MCI group and the HC group. Analysis reveals significantly less cortical thickness in the left caudal anterior cingulate (p=0.041), left lateral occipital (p=0.009) and right superior temporal (p=0.047) areas in the MCI group after adjustment for age and education.

**Table 2:** Cortical thickness in healthy control and mild cognitive impairment (mean +/- S.D., mm, adjusted for age and education).

	Healthy Control		MCI	
	
Brain region	Left	Right	Left	Right
Caudal anterior cingulate gyrus	2.689 (0.315) *	2.599 (0.296)	2.502 (0.378)*	2.512 (0.290)
Caudal middle frontal gyrus	2.258 (0.168)	2.262 (0.148)	2.218 (0.131)	2.243 (0.145)
Cuneus	1.618 (0.125)	1.619 (0.118)	1.612 (0.125)	1.606 (0.117)
Entorthinal area	3.403 (0.392)	3.605 (0.487)	3.288 (0.340)	3.522 (0.413)
Fusiform gyrus	2.639 (0.148)	2.603 (0.156)	2.577 (0.158)	2.554 (0.188)
Inferior parietal lobe	2.164 (0.123)	2.115 (0.113)	2.142 (0.135)	2.122 (0.148)
Inferior temporal gyrus	2.695 (0.161)	2.681 (0.154)	2.613 (0.158)	2.636 (0.184)
Isthmus cingulate gyrus	2.416 (0.187)	2.302 (0.225)	2.267 (0.229)	2.195 (0.206)
Lateral occipital gyrus	1.902 (0.130)*	1.879 (0.126)	1.899 (0.152)*	1.874 (0.147)
Lateral orbitofrontal gyrus	2.522 (0.140)	2.469 (0.153)	2.510 (0.164)	2.430 (0.166)
Lingual gyrus	1.787 (0.118)	1.810 (0.087)	1.782 (0.144)	1.779 (0.167)
Medial orbitofrontal gyrus	2.283 (0.170)	2.369 (0.164)	2.289 (0.181)	2.612 (0.165)
Middle temporal gyrus	2.670 (0.172)	2.746 (0.139)	2.660 (0.142)	2.715 (0.169)
Parahippocampal gyrus	2.535 (0.230)	2.557 (0.256)	2.378 (0.303)	2.489 (0.264)
Paracentral gyrus	2.271 (0.179)	2.270 (0.158)	2.223 (0.179)	2.222 (0.158)
Pars opercularis	2.357 (0.173)	2.366 (0.135)	2.351 (0.120)	2.352 (0.142)
Pars orbitalis	2.539 (0.217)	2.509 (0.235)	2.471 (0.221)	2.494 (0.247)
Pars triangularis	2.245 (0.134)	2.279 (0.148)	2.202 (0.134)	2.213 (0.162)
Periphery calcarine	1.385 (0.878)	1.427 (0.103)	1.414 (0.123)	1.446 (0.128)
Postcentral gyrus	1.819 (0.132)	1.765 (0.104)	1.779 (0.123)	1.787 (0.118)
Posterior cingulate gyrus	2.440 (0.221)	2.395 (0.198)	2.345 (0.175)	2.325 (0.177)
Precentral gyrus	2.364 (0.151)	2.343 (0.124)	2.312 (0.136)	2.284 (0.144)
Precuneus	2.128 (0.141)	2.064 (0.119)	2.086 (0.161)	2.047 (0.141)
Rostral anterior cingulate gyrus	2.820 (0.199)	2.882 (0.248)	2.744 (0.223)	2.802 (0.286)
Rostral middle frontal gyrus	2.110 (0.137)	2.154 (0.120)	2.090 (0.141)	2.139 (0.139)
Superior frontal gyrus	2.518 (0.146)	2.540 (0.142)	2.475 (0.141)	2.503 (0.137)
Superior parietal lobe	1.884 (0.135)	1.843 (0.122)	1.863 (0.126)	1.831 (0.121)
Superior temporal gyrus	2.563 (0.146)	2.596 (0.177)*	2.491 (0.161)	2.574 (0.155)*
Supramarginal gyrus	2.298 (0.126)	2.229 (0.149)	2.219 (0.141)	2.201 (0.135)
Frontal pole	2.671 (0.263)	2.634 (0.210)	2.597 (0.256)	2.593 (0.275)
Temporal pole	3.638 (0.267)	3.759 (0.301)	3.513 (0.283)	3.625 (0.293)
Transverse temporal gyrus	2.148 (0.252)	2.106 (0.254)	2.070 (0.197)	2.107 (0.203)
Insula	2.891 (0.157)	2.879 (0.175)	2.861 (0.158)	2.800 (0.165)

* p<0.05

**Table 3:** Correlation between neuropsychological performance and cortical thickness in mild cognitive impairment.

	CMMSE		CDR-Sum of boxes		ADAS-Cog		CVFT		Forward digit span		Backward Digit span	
	
Brain region	Left	Right	Left	Right	Left	Right	Left	Right	Left	Right	Left	Right
Caudal anterior cingulate gyrus	-.077	.075	-.005	.042	-.139	-.047	.041	.075	-.108	-.213	-.104	.172
Caudal middle frontal gyrus	-.202	-.171	.309	.302	-.061	.142	-.108	-.152	-.022	-.184	-.059	.018
Cuneus	-.050	.032	-.062	-.051	.062	-.097	.209	.221	-.151	-.090	.065	.120
Entorthinal area	.173	.323	-.262	-.366	*-.413	-.259	.349	.335	.101	.246	-.301	-.228
Fusiform gyrus	.213	.239	-.159	-.337	-.137	-.204	.106	.178	.156	.162	-.125	-.016
Inferior parietal lobe	.096	.029	.091	.083	.003	.058	.163	.117	-.109	-.110	-.059	.074
Inferior temporal gyrus	.191	*.508	-.023	-.198	-.216	-.369	.350	.262	.023	.214	-.102	.101
Isthmus cingulate gyrus	.336	.201	-.118	-.116	-.277	-.193	-.043	-.032	.115	.134	.120	.246
Lateral occipital gyrus	.075	-.017	-.133	.005	-.035	-.040	.085	-.016	.007	.092	.085	.225
Lateral orbitofrontal gyrus	-.028	-.040	.202	.003	-.053	-.044	.231	.125	.085	-.038	.176	-.021
Lingual gyrus	.108	.185	-.111	-.060	-.119	-.226	.172	.209	.048	.125	.165	.191
Medial orbitofrontal gyrus	.076	.046	-.023	.033	.030	-.148	.334	.376	.038	.066	.118	.047
Middle temporal gyrus	.212	.359	.084	-.182	-.025	.048	.131	.137	-.072	-.083	-.137	.180
Parahippocampal gyrus	.215	.200	*-.413	-.317	-.061	-.193	-.111	-.012	.004	.131	-.337	-.190
Paracentral gyrus	-.144	.043	.122	.197	-.073	-.044	.005	.048	-.244	-.098	.005	.054
Pars opercularis	.031	.001	.160	.101	-.031	-.117	.114	.126	-.117	.131	.028	-.187
Pars orbitalis	-.013	.059	.311	.221	.029	.099	-.175	-.050	.277	.245	.315	*.408
Pars triangularis	.045	.051	.058	-.009	-.124	-.155	.170	.159	.013	.138	.227	.302
Pericalcarine	-.029	-.251	.048	.072	-.069	-.117	.173	.194	.010	.010	.194	.038
Postcentral gyrus	-.170	-.188	.131	.107	-.013	.035	.049	.247	-.114	-.146	.042	.081
Posterior cingulate gyrus	.039	.040	-.012	.096	-.116	.057	.100	-.036	-.046	-.120	-.067	.181
Precentral gyrus	-.044	-.165	.011	.093	.012	-.088	-.040	.003	-.193	-.066	-.090	-.044
Precuneus	.102	.134	-.032	.060	-.151	-.115	.203	.184	-.021	-.106	.032	.022
Rostral anterior cingulate gyrus	-.028	-.070	.284	.239	.024	.214	.016	-.148	-.096	-.090	.087	-.014
Rostral middle frontal gyrus	-.267	-.100	.185	.116	.071	-.006	.260	*.398	.017	-.103	.053	.023
Superior frontal gyrus	-.196	-.190	.391	.255	.032	-.002	.008	.048	-.210	-.098	.007	-.119
Superior parietal lobe	.002	.029	-.024	.020	-.089	-.028	.151	.188	-.008	-.004	.128	.260
Superior temporal gyrus	.247	.232	-.142	-.242	-.089	.036	.324	.235	.086	.084	.127	.050
Supramarginal gyrus	.092	.026	.167	-.027	-.174	-.039	.175	.112	-.021	.109	.062	.149
Frontal pole	.104	.176	-.150	-.089	.042	-.080	.047	.324	.056	-.049	.323	-.005
Temporal pole	.115	.256	-.215	-.175	-.187	-.209	.356	252	.208	.085	.021	-.041
Transverse temporal gyrus	-.267	-.188	.198	.089	.131	.251	.029	-.170	-.173	-.002	.254	.156
Insula	.092	.116	.237	-.012	-.162	-.299	.276	.322	-.051	.079	.007	-.078

* p<0.01. ADAS-Cog - Chinese version of the Alzheimer’s Disease Assessment Scale-Cognitive Subscale; CDR - Clinical Dementia Rating; CMMSE - Cantonese version of the Mini-Mental State Examination; CVFT - Category Verbal Fluency Test

### Correlation between cortical thickness and neuropsychological performance in MCI group

**Figure 1.**
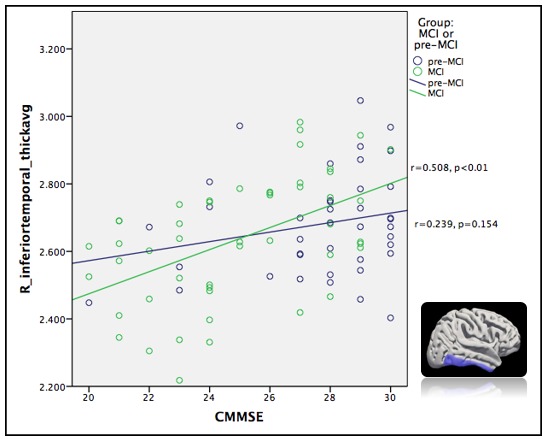
Correlation between right temporal gyrus and Cantonese version of the Mini-Mental State Examination (CMMSE).

**Figure 2.**
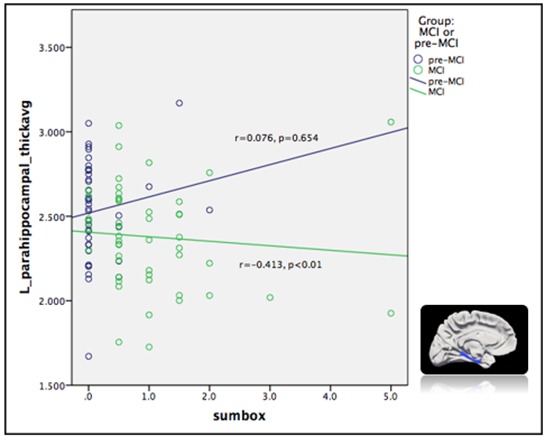
Correlation between left parahippocampal gyrus and Clinical Dementia Rating (CDR)- sum of boxes.

Almost all neuropsychological performance, except for the forward digit span, was significantly correlated with the cortical thickness (Table 3). The CMMSE score showed a significant correlation with the right inferior temporal gyrus (r=0.508; p<0.01; Fig. 1). The CDR sum of boxes score showed a significant correlation with the left parahippocampal gyrus (r=-0.413; p<0.01; Fig. 2). The performance on the ADAS-Cog showed a significant correlation with the left entorhinal area (r=-0.413; p<0.01). The CVFT score showed a significant correlation with the right rostral middle gyrus (r=0.398; p<0.01). Scores on the backward digit span test showed significant correlations with the right pars orbitalis (r=0.408; p<0.01). A thicker cortex in these regions was associated with better performance on the CVFT and on the backward digit span test.

### Correlation between cortical thickness and neuropsychological performance in HC group

Only the scores on the CVFT and the forward digit span test were found to have significant correlations with cortical thickness in the HC group (Table 4). The CVFT score showed an inverse correlation with the left middle temporal gyrus (r=-0.445; p<0.01), whilst the forward digit span test score showed a significant inverse correlation with the left pars opercularis (r=-0.496; p<0.01), the left rostral middle frontal gyrus (r=-0.422; p<0.01) and the right orbitofrontal cortex (r=-0.456; p<0.01). A thicker cortex in these regions was associated with poorer performance on the CVFT and on the forward digit span test.

## DISCUSSION

In this study, we compared the differences in cortical thickness between participants with MCI and those in an HC group. We also examined the association between neuropsychological performance and cortical thickness. The neuropsychological performance of the MCI group was significantly worse than that of the HC group, which was expected. We found significant thinning in the anterior cingulate and superior temporal regions in participants with MCI compared with those in the HC group. This result is in line with the results of previous studies [26]. It was suggested that cortical thinning begins in the temporal region and spreads to other areas [27]. In addition, the anterior cingulate region was reported in a previous study to be more sensitive comparing to other brain regions to early AD-related changes [6]. Both features were noted in our findings. The cortical thicknesses of these two areas may be useful for early identification of subjects with MCI. In addition to these two areas, the left lateral occipital region was found to be significantly thinner in the MCI group. It was relatively uncommon to note atrophy in the occipital region in subjects with MCI, but studies have nonetheless shown significant increases in the atrophy rate of the occipital region in subjects with AD and MCI [28].

**Table 4:** Correlation between neuropsychological performance and cortical thickness in healthy control.

	CMMSE		CDR-Sum of boxes		ADAS-Cog		CVFT		Forward digit span		Backward Digit span	
	
Brain region	Left	Right	Left	Right	Left	Right	Left	Right	Left	Right	Left	Right
Caudal anterior cingulate gyrus	.010	-.062	-.159	-.177	.104	.257	.131	-.166	.110	-.063	-.076	-.133
Caudal middle frontal gyrus	-.066	.116	.220	.164	.182	.106	-.031	-.043	-.314	-.253	.116	.072
Cuneus	-.060	.041	.185	.025	.169	.095	.021	.056	-.230	-.057	.155	.143
Entorthinal area	-.252	-.175	.161	.162	.272	.211	-.229	-.227	-.091	-.069	-.096	-.210
Fusiform gyrus	-.015	-.027	.122	.131	.268	.171	-.081	-.100	-.183	-.242	-.053	-.100
Inferior parietal lobe	-.055	.128	.158	-.012	.145	.176	-.024	.006	-.156	-.009	.081	.182
Inferior temporal gyrus	-.006	.239	.002	-.230	.073	-.154	-.199	-.090	-.104	-.008	.228	.013
Isthmus cingulate gyrus	-.139	-.137	.377	.249	.271	.342	-.148	-.232	-.293	-.130	.058	.247
Lateral occipital gyrus	.229	.277	-.108	-.105	.018	.088	.124	.197	.028	-.009	.259	.277
Lateral orbitofrontal gyrus	-.278	-.234	.389	.284	.153	.126	-.300	-.049	-.396	-.416	.127	.076
Lingual gyrus	-.079	.222	.105	.001	.197	.131	.122	.246	-.405	.017	-.004	.216
Medial orbitofrontal gyrus	-.191	-.068	.263	-.046	.122	.121	-.227	-.182	-.393	*-.456	-.003	.079
Middle temporal gyrus	-.125	.043	.309	.006	.248	.085	*-.445	-.306	-.195	-.133	.205	.174
Parahippocampal gyrus	-.180	-.151	.076	-.007	.109	-.029	-.293	-.201	-.241	-.235	-.046	-.131
Paracentral gyrus	.096	-.086	.036	.124	.233	.116	.130	.044	-.139	-.235	.287	.065
Pars opercularis	-.211	.117	.303	.193	.340	.153	-.094	.042	*-.496	-.258	.043	-.005
Pars orbitalis	-.228	-.187	.261	.040	-.043	.064	.064	-.045	-.225	-.355	.075	.011
Pars triangularis	-.200	-.038	.333	.207	.261	.041	-.008	-.031	-.367	-.116	-.116	-.080
Pericalcarine	-.177	-.109	.249	.097	.187	.091	.036	.020	-.237	-.331	-.040	-.093
Postcentral gyrus	.144	-.103	-.017	.057	-.011	.181	-.004	.039	-.009	-.093	.145	-.027
Posterior cingulate gyrus	-.061	-.046	.000	.064	.306	.379	-.074	-.039	-.044	-.144	.125	.095
Precentral gyrus	-.049	.016	.145	.220	.110	.183	-.031	-.015	-.326	-.183	.217	.132
Precuneus	.059	-.028	-.060	.179	.199	.170	.077	.001	-.091	-.143	.323	.204
Rostral anterior cingulate gyrus	-.108	-.117	-.111	-.086	.068	-.035	-.142	.023	-.189	-.201	-.171	-.090
Rostral middle frontal gyrus	-.276	.054	.267	.064	.037	-.003	-.181	-.092	*-.422	-.162	-.183	.247
Superior frontal gyrus	.063	-.001	.023	.158	.137	.196	-.025	-.117	-.166	-.295	.092	.088
Superior parietal lobe	.092	-.037	.092	.100	.168	.173	.039	.021	-.074	-.173	.267	.271
Superior temporal gyrus	.136	.213	.093	-.188	.202	.177	.008	-.029	-.142	.205	.011	.126
Supramarginal gyrus	.093	-.020	.134	-.105	.187	.045	-.096	-.103	-.084	-.092	.056	.038
Frontal pole	-.051	.030	-.051	-.074	.037	-.174	-.238	.074	-.132	-.086	.231	.259
Temporal pole	-.007	.131	-.037	-.069	.080	-.021	-.198	-.226	.025	.076	.105	.101
Transverse temporal gyrus	-.033	.232	.051	-.289	.131	.051	.022	.235	-.118	.214	.026	.260
Insula	.057	-.078	.038	.206	-.060	.132	-.220	-.274	-.155	-.229	-.008	-.083

* p<0.01 ADAS-Cog - Chinese version of the Alzheimer’s Disease Assessment Scale-Cognitive Subscale; CDR - Clinical Dementia Rating; CMMSE - Cantonese version of the Mini-Mental State Examination; CVFT - Category Verbal Fluency Test

### Correlation between cortical thickness and neuropsychological performance in subjects with MCI

Global cognition as measured by the CMMSE and the CDR sum of boxes showed a moderate correlation with the temporal area in participants with MCI; temporal atrophy is a hallmark of early AD-related changes. Therefore, our finding supports the notion that cortical thinning in this region is directly linked to a decline in global cognition. This may further support the use of the cortical thickness of the temporal area to predict the progression of MCI to AD.

### Difference in correlational patterns

The participants with MCI showed significant correlations between the cortical thickness in various brain areas and each of the neuropsychological performance measures, with the exception of the forward digit span test, but normal older adults showed significant correlations between cortical thickness and two neuropsychological measures only. No global cognition scores such as those on the CMMSE or the CDR sum of boxes were found to have a significant correlation with the cortical thickness in the HC group. One possible explanation for this finding is the ceiling effect of neuropsychological measures in the HC group. However, it could not explain the lack of correlation in tests such as the ADAS-Cog, CVFT and the forward digit span test, in which no prominent ceiling effects were noted. Another postulation is that the participants in the HC group had better connectivity across the whole brain and, therefore, a better compensatory mechanism. When one brain area appeared to be dysfunctional due to the loss of grey matter, other brain areas could compensate so that neuropsychological performance and global cognition are relatively maintained. In participants with MCI, due to the lower degree of connectivity across the whole brain, neuropsychological performance and global cognition directly reflected the severity of cortical thinning without compensation by other brain areas.

The second possible explanation is supported by recent research findings suggesting that MCI and AD represent a disconnection syndrome and that the cognitive impairment results from a decrease in the effectiveness of whole-brain connectivity [29, 30]. A growing body of evidence shows an alteration of functional connectivity in patients with MCI and AD, compared with health control subjects [31, 32]. The connectivity is usually increased in the local area or lobe but significantly decreased across different lobes of the brain [33]. In addition to the functional connectivity, alteration of the structural connectivity, as measured by white matter integrity, has also been reported in patients with prodromal AD [34]. Such Weakening of both functional and structural connectivity may affect the compensatory mechanism. The efficiency of the brain’s function as a single unit may then decrease. Cognitive functions become compartmentally dependent upon one or two areas and are more susceptible to degeneration and loss of neuronal cells. Further study that involves concomitant structural and functional connectivity investigation is needed to verify that the difference in the relationship between regional cortical thickness and neuropsychological performance between healthy and MCI subjects is due to changes in connectivity.

In our study, scores on the CVFT and the digit backward span test showed a positive correlation with cortical thickness in the MCI group. This means that a decrease in cortical thickness is associated with poorer performance on neuropsychological tests, which is compatible with our previous hypothesis. The neuropsychological performance may be more dependent upon the integrity of grey matter in specific brain regions in subjects with MCI due to the impairment of whole-brain connectivity. However, the HC group members had the opposite result: the CVFT and the forward digit span test scores showed a negative correlation with cortical thickness, which means that an increase in cortical thickness is associated with poorer performance on neuropsychological tests. The Previous study also showed that the positive correlation between brain volume and cognition was not found in healthy subjects [35]. One of the possibilities is that the neuropsychological tests were not sensitive enough to reflect the changes in the preclinical phase. The healthy subjects may have AD pathology without symptoms. The previous study found neuronal hypertrophy in the hippocampus and anterior cingulate gyrus neurons among asymptomatic AD patients compared with MCI and control, which may be due to compensation at the local level [36]. Such local compensation may increase cortical thickness but have limited effect on the neuropsychological performance, causing the negative correlation between cortical thickness and neuropsychological performance. However, we could not confirm this explanation in the current study without measurement of AD pathology in our subjects.

Most cognitive training targets deficits in individual cognitive domains. For example, if someone was noted to have a memory problem, the most direct treatment would be to train the memory domain only. However, the effectiveness of this kind of training is in doubt [37]. The effects of training are often short-lived, and the improvement does not translate to daily functions. This phenomenon may be explained by the theory of the disconnection syndrome. The impairment of cognition is due to the connectivity problem rather than solely due to the loss of function of the individual brain areas responsible for that cognitive function. If this is really the case, the aim of cognitive training should be to enhance brain connectivity instead of training up individual cognitive domains. Such connectivity training may have longer and better effects and could likely be generalised to improvement in daily functioning. Further study is needed to demonstrate this conceptual idea.

### Limitations of study

There were a few limitations of this study. First, the sample size was relatively small and may result in under-power of the current study to detect the difference between the groups. Besides, the pattern difference in correlation between neuropsychological performance and the cortical thickness between MCI and HC groups was mainly descriptive instead of the direct statistical result in the current study. Further study with larger sample size would be needed in order to perform the direct statistical test for comparing the correlation between two groups because a significant amount of multiple comparisons would be involved. Another limitation of our study is the significant difference in education level and age between the MCI group and the HC group; the participants in the MCI group were older and had lower education levels. Comparison of the two groups and correlational analysis were performed with education and age as co-variates to minimise the effect of a baseline difference between the two groups. At last, we had not done the familywise correction for the cortical thickness comparison, which may increase the chance of the false positive result in the current study.

### Conclusions

Our findings suggest that the MCI group had significant thinning over the right temporal, left anterior cingulate and left lateral occipital regions compared with the HC group. Cortical thinning in the temporal region was associated with the global cognition change in participants with MCI and may be useful to predict the progression of MCI to AD. The different pattern between the MCI group and the HC group in the correlation of cortical thickness to neuropsychological performance may be explained by the hypothesis of MCI as a disconnection syndrome. Further imaging studies such as resting state and diffusion tensor imaging are warranted to investigate the alteration in functional and structural connectivity in subjects with MCI. Treatment for cognitive impairment should be directed to the enhancement of brain connectivity in view of the role that a disconnection problem plays in cognitive decline.
